# Anthrax Lethal Toxin Induced Lysosomal Membrane Permeabilization and Cytosolic Cathepsin Release Is Nlrp1b/Nalp1b-Dependent

**DOI:** 10.1371/journal.pone.0007913

**Published:** 2009-11-18

**Authors:** Kathleen M. Averette, Matthew R. Pratt, Yanan Yang, Sara Bassilian, Julian P. Whitelegge, Joseph A. Loo, Tom W. Muir, Kenneth A. Bradley

**Affiliations:** 1 Department of Microbiology, Immunology & Molecular Genetics, University of California Los Angeles, Los Angeles, California, United States of America; 2 Laboratory of Synthetic Protein Chemistry, The Rockefeller University, New York, New York, United States of America; 3 Department of Chemistry and Biochemistry, University of California Los Angeles, Los Angeles, California, United States of America; 4 The Pasarow Mass Spectrometry Laboratory, The NPI-Semel Institute, David Geffen School of Medicine, University of California Los Angeles, Los Angeles, California, United States of America; Columbia University, United States of America

## Abstract

NOD-like receptors (NLRs) are a group of cytoplasmic molecules that recognize microbial invasion or ‘danger signals’. Activation of NLRs can induce rapid caspase-1 dependent cell death termed pyroptosis, or a caspase-1 independent cell death termed pyronecrosis. *Bacillus anthracis* lethal toxin (LT), is recognized by a subset of alleles of the NLR protein Nlrp1b, resulting in pyroptotic cell death of macrophages and dendritic cells. Here we show that LT induces lysosomal membrane permeabilization (LMP). The presentation of LMP requires expression of an LT-responsive allele of *Nlrp1b*, and is blocked by proteasome inhibitors and heat shock, both of which prevent LT-mediated pyroptosis. Further the lysosomal protease cathepsin B is released into the cell cytosol and cathepsin inhibitors block LT-mediated cell death. These data reveal a role for lysosomal membrane permeabilization in the cellular response to bacterial pathogens and demonstrate a shared requirement for cytosolic relocalization of cathepsins in pyroptosis and pyronecrosis.

## Introduction

The innate immune system is the first defense against invading microorganisms, and functions to either clear or limit infection until an adaptive response can be mounted. Innate immune cells recognize pathogen-associated molecular patterns through pattern recognition receptors such as toll-like receptors and nucleotide oligomerization domain-like receptors (NLRs) [1], [2]. NLRs identify cytosolic danger signal(s) or foreign molecules and activate protective cellular responses. Some NLRs bind directly, or indirectly, to caspase-1 (GeneID: 12362) within large molecular weight complexes called inflammasomes, and facilitate activation of caspase-1 resulting in processing and release of the proinflammatory cytokines IL-1β (GeneID: 16176) and IL-18 (GeneID: 16173) [3]. Multiple types of inflammasomes exist that vary by the NLR that activates formation (e.g. NLRP3-inflammasome, Nlrp1b-inflammasome, etc.). In macrophages and dendritic cells (DCs), NLR-induced inflammasome activation can lead to pyroptosis, a newly described necrosis-like programmed cell death [4].

Pyroptosis is induced in response to numerous pathogens including Shigella, Salmonella, Listeria, Legionella, Pseudomonas, Mycobacterium, Yersinia, Burkholderia, and bacterial products flagellin and *B. anthracis* lethal toxin (LT) [1], [4]–[9]. *B. anthracis* LT is produced during infection and typically functions to suppress innate immunity [10]–[12]. The NLR family member *Nlrp1b* (also known as *Nalp1b*; GeneID: 637515) recognizes the activity of *B. anthracis* LT in the host cytosol, but is highly polymorphic in mice with only a subset of alleles conferring a pyroptotic response to LT [9]. Macrophages that express an LT-sensitive allele of *Nlrp1b* (LT^S^) undergo pyroptosis in the presence of this toxin, releasing inflammatory cytokines that activate innate immunity [9], [13]. It is not understood how Nlrp1b controls recognition of LT or what downstream events lead to cell death [1], [7]. Here we used LT to investigate the mechanism of cell death that occurs during pyroptosis.

LT is secreted by *B. anthracis* as two proteinaceous subunits, protective antigen (PA; GeneID: 2820165) and lethal factor (LF; GeneID: 2820148) [14]. The binding subunit, PA, attaches to host cell receptors and oligomerizes to form a binding site for the catalytic subunit, LF [15]–[18]. PA-LF complexes are endocytosed and trafficked to acidic vesicles, where PA forms a membrane pore and translocates LF into the cytosol [18]. LF is a zinc-dependent metalloproteinase that cleaves the N-terminus of mitogen activated protein kinase kinases (MKKs) 1–4, 6, and 7 [19], [20]. Cleavage of MKKs by LT occurs at or near MKK-MAPK binding sites, disrupting downstream MAPK signaling [21], [22]. Although disruption of MAPK signaling alters numerous signaling pathways and transcription, the activating danger signal(s) that induce pyroptosis are unknown.

Lysosomal membrane permeabilization (LMP), the loss of proton gradients in acidic compartments and leakage of lysosomal proteins into the cytosol, is associated with both apoptosis and necrosis [23]–[28]. Severe LMP, characterized by rapid loss of lysosomal membrane stability, is primarily associated with the final stages of necrosis while mild LMP, or slow leakage of lysosomal contents, alters cellular signaling and can induce caspase-dependent apoptosis or caspase-independent apoptosis-like cell death [24], [27], [29], [30]. A role for LMP in LT-mediated pyroptosis was recently described [31]. We provide confirmatory evidence that LMP occurs during LT-mediated pyroptosis and reveal that LMP is dependent on the presence of an LT-responsive Nlrp1b.

## Results

### Acidic compartments are compromised during LT-induced pyroptosis

**Figure 1:**
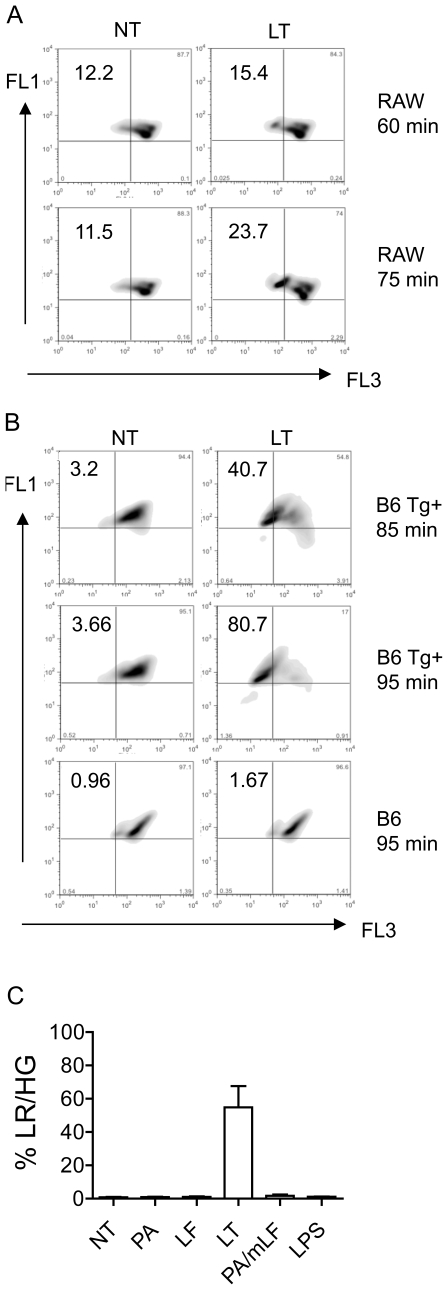
LT causes LMP in LT^S^ macrophages. (A) RAW 264.7 (RAW) cells pretreated with AO and subjected to LT (3 µg/mL LF and 1 µg/mL PA) for 60 or 75 minutes or media alone (NT). Cells were analyzed by flow cytometry, live cells were gated based on forward and side scatter and cells were analyzed for red (FL3) and green (FL1) fluorescence. Cells are depicted here as a density plot. Upper left quadrants represent cells with low red and high green fluorescence (LR/HG). Numbers correspond to percent of cell population in LR/HG quadrant. (B) C57BL/6*^Nlrp1b(129S1)^* BMDMs (B6 Tg^+^) or littermate controls (B6) were pretreated with AO and subjected to either LT (1 µg/mL LF and 1 µg/mL PA) for 85 or 95 minutes or media alone (NT). Cells were analyzed as in (A). Density plot represent BMDMs from one of three C57BL/6*^Nlrp1b(129S1)^* or C57BL/6 littermate controls and are representative of results obtained. (C) C57BL/6*^Nlrp1b(129S1)^* BMDMs were treated with 1 µg/mL of LF, PA, LF and PA (LT), PA and LF-H719C (PA/mLF), or 10 ng/mL of lipopolysaccharide (LPS) for 90 min. Cells were collected and analyzed for red and green fluorescence as in (A). BMDMs from three C57BL/6*^Nlrp1b(129S1)^* were used for each condition and error bars represent standard deviation.

A hallmark of LMP is the loss of lysosomal acidity. To determine if lysosomal pH is affected by LT, we analyzed macrophages for alterations in acridine orange (AO) staining following toxin challenge. AO is a cell permeable, lysosomotropic dye that is protonated and sequestered within acidic compartments such as late endosomes and lysosomes. The fluorescence emission of AO is concentration dependent, such that at high concentrations (e.g. in lysosomes) it fluoresces red, while under diffuse conditions (e.g. in the cytosol) it fluoresces green. LMP can be recognized by a decrease in red AO fluorescence while maintaining high green AO fluorescence. RAW 264.7 cells, a murine macrophage-like cell line that expresses LT^S^ alleles of *Nlrp1b*, were pre-loaded with AO and treated with or without LT for various incubation times then analyzed by flow cytometry. In LT treated cells, there was a significant increase in a subpopulation of cells that emit low red and high green (LR/HG) fluorescence compared to control-treated cells (Figure 1A) and this population increased over time following LT challenge. Of note, a separate population of cells appeared that displayed both low red fluorescence and low green fluorescence compared to the entire population (Figure S1). The loss in fluorescence in both channels is consistent with loss of cell membrane integrity resulting in the combined loss of acidic compartments and cytoplasmic contents containing AO. Indeed, the number of cells in this population was proportional with duration of LT treatment and analysis of forward versus side scatter profiles is consistent with non-viable cells.

Next, we tested whether appearance of the AO LR/HG subpopulation depends on *Nlrp1b* allelic variations. RAW 264.7 cells are derived from BALB/c mice which express LT^S^ *Nlrp1b*, whereas IC-21 macrophage-like cells are derived from LT-resistant (LT^R^) *Nlrp1b* expressing C57BL/6 mice and do not undergo pyroptotic death in response to LT. IC-21 cells showed no increase in LR/HG population in response to LT (Figure S2A). To directly test whether *Nlrp1b* allelic differences were sufficient to explain differential AO staining, we tested bone marrow derived macrophages (BMDMs) derived from C57BL/6 mice expressing a transgenic LT-responsive *Nlrp1b* allele from 129S1 mice (C57BL/6*^Nlrp1b(129S1)^* mice; Tg^+^), or littermate controls (Tg^−^). C57BL/6 Tg- BMDMs showed no change in geometric mean fluorescence when subjected to flow cytometry following AO staining and LT treatment (Figure 1B). However, C57BL/6*^Nlrp1b(129S1)^* Tg^+^ BMDMs showed a time-dependent shift into LR/HG following LT-treatment (Figure 1B). Thus, in both BMDMs and immortalized macrophage-like cell lines, LT causes relocalization of AO that is dependent on expression of an LT-responsive *Nlrp1b* allele.

During intoxication, PA forms cation-selective, ion-conducting channels in endosomal membranes that translocate LF in a voltage-dependent manner [18]. To determine if the LR/HG population observed in response to LT was due to PA pore formation rather than LMP, we performed AO staining of cells treated with PA alone or PA in the presence of a catalytically inactive lethal factor, LF-H719C, which binds but does not cleave MKKs [32]. We observed a pronounced increase in LR/HG only in cells treated with the catalytically active LF and PA in both C57BL/6*^Nlrp1b(129S1)^* BMDMs (Figure 1C) and RAW 264.7 cells (data not shown). Therefore, alterations in AO staining in response to LT are not explained by PA pore formation, but rather require the catalytic activity of LF.

**Figure 2:**
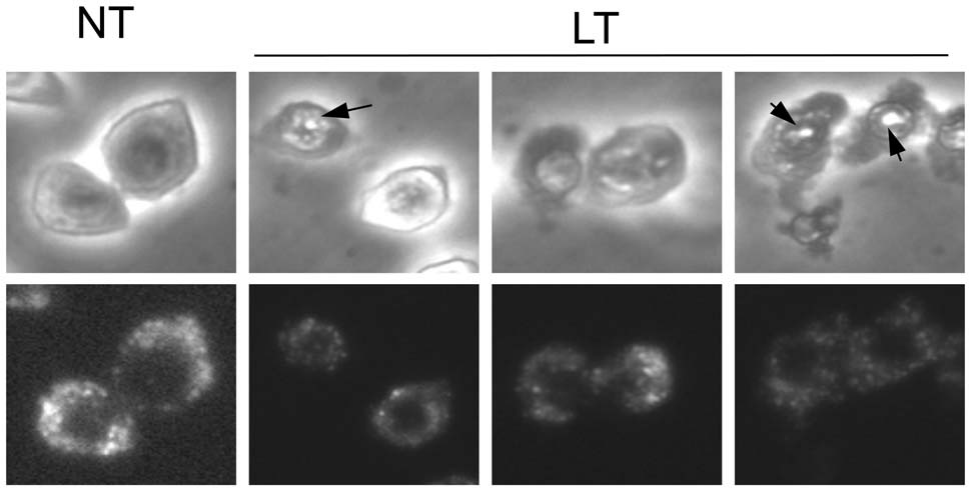
Lysosome ultrastructure appears unaltered during Nlrp1b-mediated pyroptosis. (A) RAW 264.7 cells were pre-stained with Lysotracker Red DND-99 followed by LT or untreated (NT) for 75 min and imaged on glass slides at 40× magnification. Black arrows correspond to condensed nuclear DNA observed in pyroptotic cells.

A measured decrease in acidic compartments could be due to LMP or from loss of acidic vesicles through exocytosis, macroautophagy or disintegration of lysosomal membranes. To determine if LT-treated cells show signs of lysosomal loss or fusion, we stained and visualized acidic vesicles with Lysotracker Red, a fluorescent probe that associates with the membranes of acidic compartments, but unlike AO, continues to stain de-acidified lysosomes. When added prior to a cytotoxic stimulus, Lysotracker Red will continue to stain lysosomal membranes that undergo LMP, but will disperse throughout the cell if the lysosomal membrane disintegrates or fuses with the plasma membrane [33], [34]. Likewise, fusion of lysosomal membranes with other compartments would result in a decrease in fluorescence intensity or an increase in acidic vesicle size [35], [36]. In LT treated cells, Lysotracker Red-stained lysosomes were clearly visible following LT-treatment up to the time of death (Figure 2). Interestingly, Lysotracker Red continued to stain lysosomal membranes up to 30 min following cell death, as determined by membrane permeability to trypan blue (data not shown). This observation is consistent with LMP, in which lysosomes lose acidity and release lysosomal contents but appear structurally normal [24], [37]. Furthermore, no significant increase in lysosome size was observed following LT treatment, indicating that macroautophagy does not occur under the assay conditions employed here (Figure 2) [38], [39]. Therefore, the increase in the AO LR/HG population following LT treatment is not due to major lysosomal exocytosis or lysosomal fusion with non-acidic vesicles, but is consistent with LMP.

### Cathepsin B is active in the cytosol and inhibition of cathepsins blocks LT-mediated pyroptosis

**Figure 3:**
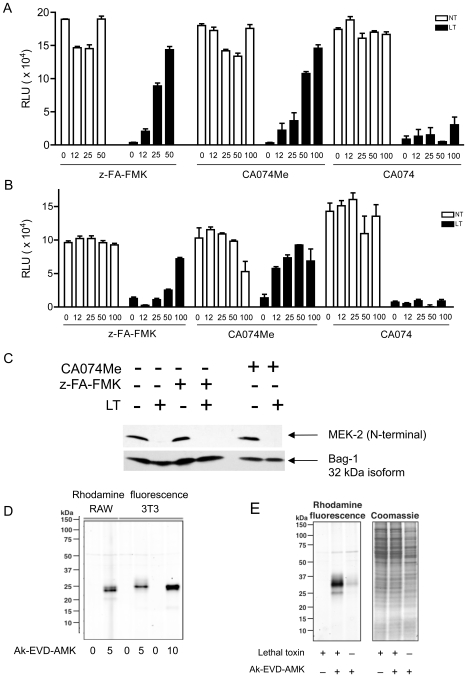
CtsB is active in the cytosol and inhibition of cathepsin activity blocks LT-mediated pyroptosis. (A) C57BL/6*^Nlrp1b(129S1)^* BMDMs were pre-treated with varying concentrations of z-FA-FMK, CA074Me, or CA074 for 4 hours, followed by addition of 400 ng/mL of PA and 300 ng/mL of LF (LT), or no treatment (NT), for an additional 3.5 hours. Cytotoxicity was measured using ATP-lite. BMDMs from three C57BL/6*^Nlrp1b(129S1)^* were used with each condition tested in quadruplicate. Error bars represent standard deviation. (B) RAW 264.7 cells were pre-treated with varying concentrations of z-FA-FMK, CA074Me, or CA074 for 4 hours, followed by addition of 400 ng/mL of PA and 300 ng/mL of LF (LT), or no treatment (NT), for an additional 3.5 hours. Cytotoxicity was measured using ATP-lite. The data presented here are representative of three or more independent experiments. Each point represents the mean of triplicate or quadruplicate samples from a single experiment, with error bars representing standard error. (C) RAW 264.7 cells were pre-treated with 100 µM z-FA-FMK or 50 µM CA074Me for 4 hours followed by LT (400 ng/mL PA and 300 ng/mL LF; +) or no toxin (−) for 2.5 hours. Cell lysates were subjected to western blot analysis and probed with an antibody that recognizes the N-terminus of MEK2. (D) Specificity of the ctsB probe Ak-EVD-AMK was determined by treating RAW 264.7 or NIH 3T3 cells with or without high concentration (5 or 10 µM) of probe. Cycloaddition assays were performed on Ak-EVD-AMK labeled cellular lysates with azido-rhodamine, and Ak-EVD-AMK labeled proteins were analyzed by in-gel fluorescence. The pro-form of ctsB is 43 kDa, whereas active ctsB is seen as either 31 kDa or 25 kDa. (E) In-gel fluorescence showing electrophoretically separated proteins from RAW 264.7 cells treated with 400 ng/mL of PA and 300 ng/mL of LF and/or, low dose (625 nM) Ak-EVD-AMK. Under these conditions, cytosolic ctsB is preferentially labeled. Cycloaddition was preformed as in (D). The gel was then stained with coomassie to indicate equal sample loading.

Cathepsins are a subtype of lysosomal acid hydrolases that participate in protein turnover, antigen processing, pro-hormone activation and, when released into the cytosol, cell death [40]. Cathepsin B (ctsB) activity was reported to be required for LMP-mediated apoptosis and necrosis in response to multiple insults including TNF-α, the chemotherapeutic pyrimethamine, the antibiotics nigericin and staurosporine and the *Mycobacterium tuberculosis* vaccine Bacillus Calmette-Guerin [41]–[46]. We tested whether cathepsin proteolytic activity is required for LT-induced cytotoxicity. Using CA074Me and z-FA-FMK, two compounds that inhibit cathepsins including ctsB and ctsL, we observed that both C57BL/6*^Nlrp1b(129S1)^* BMDMs and RAW 264.7 cells were protected from LT (Figure 3A and 3B). In addition to their cytosolic roles in cell death, cathepsins function within the lysosomal lumen and extracellularly. To differentiate between effects of CA074Me on intracellular versus extracellular cathepsin activity, we utilized CA074, a ctsB inhibitor that is not cell membrane permeable. Neither C57BL/6*^Nlrp1b(129S1)^* BMDMs, nor RAW 264.7 cells, were protected from LT by pretreatment with CA074 (Figure 3A and 3B). Therefore, the protection afforded by CA074Me and z-FA-FMK is from inhibition of intracellular cathepsin activity.

To ensure that cathepsin inhibitors did not negatively impact LT entry or activity, LF activity was analyzed by probing for MEK2 cleavage following LT challenge. No detectable change in LF activity was observed in the presence of z-FA-FMK (Figure 3C). Although a slight delay in MEK1 cleavage was detected in cells pretreated with 100 µM CA074Me (data not shown), no defect in LF activity was apparent at 50 µM (Figure 3C), a concentration that still provided maximal protection from LT (Figure 3B). Therefore, inhibition of intracellular cathepsin activity blocks LT-mediated cell death.

To directly test if ctsB is active in the cytosol during LT-mediated cell death, we utilized a novel ctsB-specific molecular probe, Ak-EVD-AMK [47]. This probe specifically reacts with active but not the pro-form of ctsB by covalently modifying the active site cysteine of this protease. Ak-EVD-AMK reacts with both cytosolic and lysosomal ctsB at high concentrations (5-10 µM) (Figure 3D), but is specific for cytosolic ctsB at lower concentrations (625 nM). Ak-EVD-AMK contains an alkyne functional group that allows for subsequent covalent modification of labeled proteins with azide-containing imaging probes via a copper catalyzed cycloaddition reaction. RAW 264.7 cells were treated with LT, labeled with low dose Ak-EVD-AMK to detect cytosolic ctsB, lysed and the alkyne-modified proteins were detected by cycloaddition of azido-rhodamine followed by SDS-PAGE and direct in-gel fluorescence detection (Figure 3E). A strong increase in Ak-EVD-AMK labeling of ctsB was observed at low probe concentration in LT treated cells compared with no-toxin controls. Thus, LT induces ctsB release into the cytosol, consistent with LMP. Taken together, our data indicate that ctsB is active in the cytosol during LT-mediated pyroptosis and that cathepsins are required to induce cell death.

### LT-induced LMP is a late event in pyroptosis

**Figure 4:**
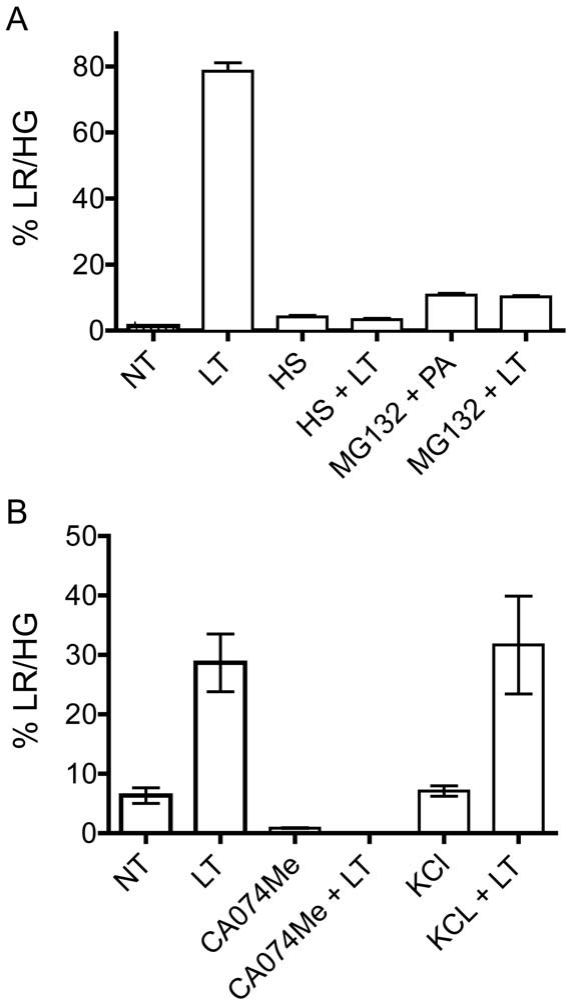
Heat shock, proteasome and ctsB/L inhibition, but not potassium chloride, prevents LT-induced LMP. (A) C57BL/6*^Nlrp1b(129S1)^* BMDMs were heat shocked at 42°C (HS) for 15 min prior to addition of LT (1 µg/mL of PA and 500 ng/mL of LF) for 90 min. Proteasome inhibition was accomplished with co-incubation of cells with 10 µM MG-132 and either LT (1 µg/mL of PA and 500 ng/mL of LF) or PA only for 90 min. Cells were collected for flow cytometry and % LR/HG was determined as in Figure 1A. Experiments were preformed using BMDMs from three C57BL/6*^Nlrp1b(129S1)^* and samples were collected in triplicate. Error bars represent standard deviation. (B) In a separate experiment C57BL/6*^Nlrp1b(129S1)^* BMDMs were pretreated with LT (400 ng/mL PA and 300 ng/mL LF) or without toxin (NT) for 2.5 hours. Cells were also either pre-incubated with 50 µM CA074Me for 4 hours or co-treated with 150 mM potassium chloride (KCl) followed by LT. Cells were collected for flow cytometry and % LR/HG was determined as in Figure 1A. Experiments were preformed using BMDMs from three C57BL/6*^Nlrp1b(129S1)^* and samples were collected in triplicate. Error bars represent standard deviation.

Several recent studies have begun to elucidate the pyroptotic response to LT. Events that occur following intoxication include (in order of occurrence) cleavage of MEKs and/or an unidentified target(s), proteasome cleavage of unknown target(s), mitochondrial dysfunction, potassium efflux, caspase-1 inflammasome activation, plasma membrane permeability and cellular lysis with release of IL-1β and IL-18 [9], [13], [48]–[52]. To determine the stage at which LMP contributes to pyroptosis, we performed epistasis experiments using chemical inhibitors and conditions that prevent LT-induced pyroptosis. The observation that LMP is not detected in LT-treated wildtype C57BL/6 BMDMs nor IC-21 cells (Figure 1B and 2A) suggests that LMP is downstream of Nlrp1b activity. We found that heat shock or the presence of proteasome inhibitors, two conditions that inhibit LT-induced caspase-1 activation and pyroptosis [48], [49], [51], [53], [54], prevented a shift to LR/HG in C57BL/6*^Nlrp1b(129S1)^* BMDMs (Figure 4) or RAW 264.7 cells (Figure S2). Interestingly, the presence of 150 mM exogenous potassium chloride, conditions that protect macrophages from LT lysis (data not shown), did not prevent AO relocalization (Figure 4B). Of note, potassium chloride protection from LT-mediated cellular lysis is also downstream of disruption of mitochondrial membrane potential [48]. Thus, our data supports potassium efflux as a late event in pyroptosis.

### Cellular stress proteins are altered during LT-mediated pyroptosis

LT-induced pyroptosis appears to be independent of gross transcriptional changes [55]-[57], and is likely governed by changes in the proteome. We investigated the affect of LT on the macrophage proteome using two-dimensional difference gel electrophoresis (2D-DIGE). MEK1 was cleaved by 20 min, MEK2 by 40 min and cellular lysis occurred between 75 and 90 min post-LT challenge under the conditions employed here (data not shown). We found generalized proteolysis following 70 min of LT intoxication (data not shown), consistent with LMP or extrinsic apoptosis [26]. Since we detected LF cytosolic activity by 20 min, we chose to analyze the proteomic changes following LT challenge at 30 and 40 min post-LT to identify early events in the pyroptotic death pathway. LF-specific events were further elucidated by comparison with macrophages treated with the binding component (PA) alone.

**Figure 5:**
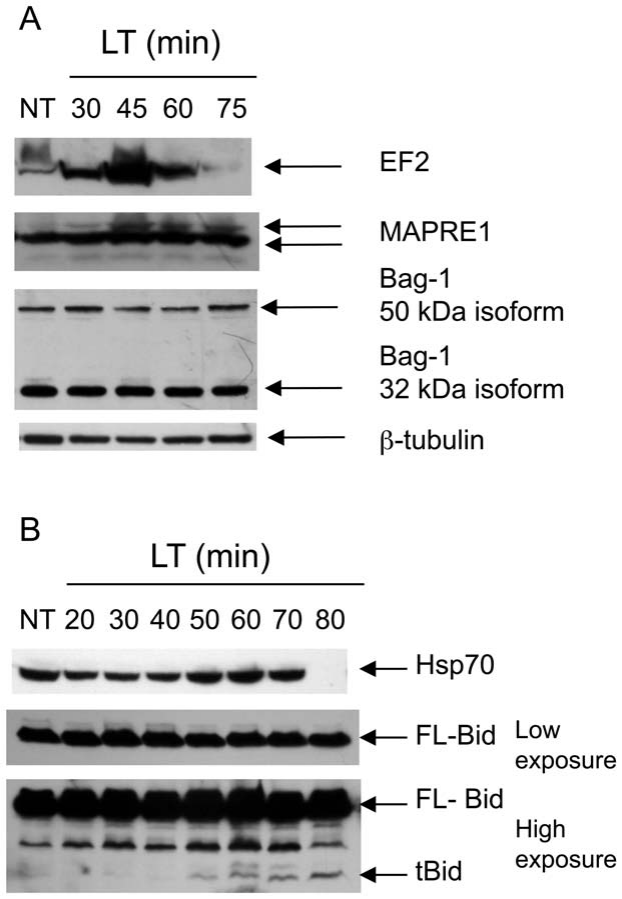
Proteomic changes in LT-treated cells. Western blots showing electrophoretically separated proteins from RAW 264.7 cells treated with LT for various time points, or untreated (NT). Arrows indicate protein isoforms detected using protein-specific primary antibodies and fluorescently labeled secondary antibodies. (A) Identical cellular lysates were subjected to SDS-PAGE, transferred to PVDF and probed with different primary and secondary antibodies. β-tubulin was used as an equal loading control in each experiment. This blot represents one of three independent experiments showing similar results. (B) Identical cellular lysates were subjected to SDS-PAGE, transferred to PVDF and probed with different primary and secondary antibodies. Anti-Bid antibody recognizes both full-length Bid (FL-Bid) and the truncated form (tBid). The membrane was exposed to film for 1 sec (low exposure) or 45 sec (high exposure) to detect both FL-Bid and tBid. tBid protein surfaces at 50 min following LT treatment at high toxin concentrations. This blot represents one of three independent experiments showing similar results.

2D-DIGE identified several proteins whose abundance increased or decreased following LT challenge. The identities of proteins whose abundance changed most significantly were determined by excising protein spots, followed by trypsin digestion and mass spectrometric analysis (Table 1). Proteome changes were validated by western blot analysis, which confirmed that microtubule-associated protein, RP/EB family, member 1 (Mapre1; GeneID: 13589), eukaryotic translation elongation factor 2 (EF-2; GeneID: 13629) (Figure 5A) and heat shock protein 70 kDa (Hsp70; GeneID: 15511) (Figure 5B) increase following LT challenge. Interestingly, we see fluctuations in protein abundance followed by loss of both Hsp70 and EF-2 at later intoxication time points (Figure 5A and 5B). Bcl-2-associated athenogene 1 (Bag-1; GeneID: 12017) is an anti-apoptotic gene that associates with Mapre1, Hsp70 and nuclear hormone receptors [58]. We did not, however, observe changes in Bag-1 protein levels following LT challenge (Figure 5A). Lamin A (GeneID: 16905) appears to be processed during LT-treatment since we found Lamin A to decrease in one excised spot while increasing in another. We also found alterations in levels of α-enolase, a known substrate of caspase-1 that colocalizes with Nlrp1b inflammasomes [13], [59]. Interestingly, cathepsin 7 precursor (also known as CTS1 and cts7; GeneID: 56092) is homologous to ctsL but is understudied and primarily associated with embryonic development. Cathepsin 7 is expressed in RAW 264.7 cells and may be proteolytically activated or change subcellular localization in response to LT. Of the proteins found to change following LT treatment, the vast majority are activated by or involved with the cellular stress or heat shock response (Table 1). In addition to inflammasome formation, it appears that LT activation of Nlrp1b causes a stress response that results in numerous changes in the proteome followed by generalized proteolysis.

**Table 1:** Proteomic changes that occur in RAW 264.7 cells treated with LT.

Accession Protein ID	Protein name	Stress (*)	I/D
NP_034608	Hsp70	*	I
NP_112442	Hsc70	*	I
NP_062412	Cathepsin 7 precursor		I
NP_075608	α-enolase	*	I
NP_031933	EF-2	*	I
NP_598890	SNEV	*	I
NP_031922	Mapre1	*	I
NP_001002011	Lamin A	*	I/D
NP_079683	M16-peptidase/putative ubiquinol-cytochrome c reductase core protein 1		I
NP_080405	ERp29c	*	I
NP_542364	Nuclear aco2		D
AAA40075	Ribosomal protein S4		D
NP_001002011	Lamin A	*	D
BAA01862	p66 mot1	*	D
NP_034860	annexin A1	*	D

Proteins whose abundance was altered at both 30 and 40 min post-LT challenge in 2D-DIGE were identified through LC-MS/MS. Stars represent proteins whose abundance change is recorded in the literature to occur following heat shock or stress. Proteins that increased (I) following exposure to LT, versus PA only, and those that decreased (D) relative to PA only are indicated. Fold increase or decrease varied from 0.5 to 3.0.

**Figure 6:**
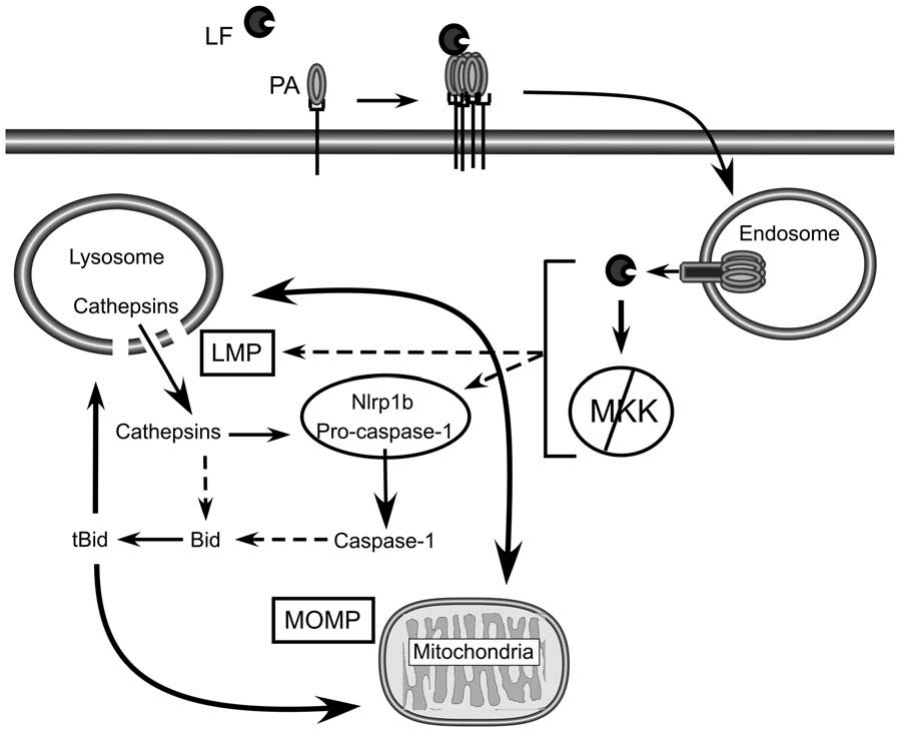
Model of LF internalization and activation of LMP and MOMP. LF binds oligomerized PA pre-pore and is internalized into endosomes where acidic pH triggers PA to form a pore in the endosomal membrane and translocate LF into the host cytosol. Following translocation, LF cleaves MKKs and induces Nlrp1-dependent pyroptosis. LF directly, or indirectly, causes LMP, resulting in release of cathepsins into the cytosol. Cathepsins or LMP-mediated signaling may directly activate the inflammasome. Alternatively, LMP may occur downstream of inflammasome activation, potentially through caspase-1 mediated cleavage of Bid. Activation of caspase-1 or cytosolic release of cathepsins can result in cleavage of Bid and a positive feedback amplification of LMP, inflammasome activation and mitochondrial outer membrane permeabilization (MOMP).

Finally, we also found that Bid (GeneID: 12122), a potential mediator of LMP-mediated cell death [24], [60], [61], is processed to its active form, tBid, in the presence of LT (Figure 5B). Interestingly, Bid can be cleaved by cathepsins B, H, L, S, K [30], [62], and potentially caspase-1 [63]. Therefore, Bid may amplify an LMP positive feedback loop (Figure 6A) and is a potential mediator of LT-induced pyroptosis. Our data supports pyroptosis as a type of programmed cell death mediated by Nlrp1b, cathepsins and potentially Bid.

## Discussion

Pyroptosis is a pro-inflammatory PCD that occurs in response to cellular recognition of danger signals [1]–[3], [64]. Although requiring caspase-1, pyroptosis maintains characteristics of necrosis [65], [66], a caspase-independent cell death. Here we show that LMP occurs during LT-mediated pyroptosis, which leads to the release of ctsB into the cytosol and resultant cell death. A causal role for cytosolic cathepsin activity in pyroptosis is indicated by the ability of CA074Me and z-FA-FMK, but not CA074, to block LT-mediated cytolysis. Lysosomal membranes can be observed up to and after the time of plasma membrane permeability, suggesting that LMP, rather than lysosomal exocytosis, disintegration or fusion, occurs during LT-mediated pyroptosis.

Previously, pyroptosis was differentiated from pyronecrosis by the requirement for caspase-1 and ctsB, respectively [8]. Although *casp-1* ^−/−^ macrophages show reduced sensitivity to LT, they are partially susceptible to LT-mediated cytolysis by an unknown process [9]. In the absence of caspase-1 activity, other mediators of LMP and pyroptosis, such as cathepsins, may be sufficient to induce cell death. Since ctsB can induce cell death similar to pyroptosis in the absence of caspase-1 [8], the role of cathepsins in pyroptosis may be substantial.

Inflammasome complexes are typically comprised of caspase-1, caspase-11, a NOD/NLR family member and a caspase adaptor protein, ASC. Unlike other NLRPs, Nlrp1b lacks a pyrin domain, the ASC-binding domain, but does encode a caspase recruitment domain (CARD) that may directly bind caspase-1 [9]. In support of an ASC-independent role in Nlrp1b-mediated cell lysis, RAW 264.7 cells, which lack ASC expression [67], respond to LT similarly to ASC-expressing J774A.1 macrophage cell line and C57BL/6*^Nlrp1b(129S1)^* BMDMs (Figure 1) [9], [68], [69]. Further, size exclusion chromatography of J774A.1 cells treated with LT revealed a shift in localization of caspase-1 from a low-molecular weight fraction containing ASC to a high molecular weight fraction containing Nlrp1b, but not ASC [13]. Interestingly, ASC is required for IPAF/NLRC4-mediated caspase-1 activation and IL-1β production, but is not required for IPAF-mediated pyroptosis [70]–[72]. Finally, ASC is not required for human NALP1 inflammasome activation [73]. Therefore, ASC is not likely required for LT-mediated cell lysis, but may enhance IL-1β and IL-18 release in response to this toxin. The role, or lack thereof, for ASC in Nlrp1b-mediated pyroptosis is currently being pursued in our laboratory.

There are conflicting data on exactly where in the cell death pathway LMP plays a role. On one hand, we find that LMP, like mitochondrial outer membrane permeability (MOMP), occurs after involvement of Nlrp1b and the proteasome, indicating that this is a late event. Furthermore, heat shock prevents LMP and protects macrophages from lysis even when applied late in the intoxication process [51]. In contrast, we observed that potassium chloride, which blocks inflammasome activation in other systems [74], did not block LMP, suggesting that LMP occurs prior to or independent of inflammasome activation. Interestingly, Newman *et al.* found that the potassium channel inhibitor quinidine did not prevent LT-induced LMP [31], however data is not shown. Although preventing cellular lysis, 150 mM KCl supplemented media does not inhibit LT-induced MOMP [48]. Since mitochondrial membrane disruption induces LMP [75], we hypothesize that LT-induced MOMP is sufficient to induce LMP in an Nlrp1b-independent manner. It would be interesting to determine if quinidine prevents LT-induced MOMP, thus preventing LMP.

We propose a model whereby LMP participates in a positive feedback loop that requires Nlrp1b to amplify an LT-mediated danger signal (Figure 6). Precedence for such a positive feedback loop exists. For example, cathepsins released into the cytosol during LMP cleave the pro-apoptotic protein Bid that induces MOMP and further LMP [25], [26], [75]. Cathepsins also cleave and activate caspase-1 [76], and both cathepsins and caspase-1 cleave Bid [30], [60], [62], [63], further augmenting a positive feedback amplification loop. In this model, LMP could be up- or downstream of inflammasome activation. Indeed, LMP can itself act as a danger signal, inducing inflammasome activation and cell death through changes in calcium concentration, cytosolic cathepsin activity, oxidative stress, or induction of MOMP [77]–[79]. It is possible that initial LMP occurs upstream of Nlrp1b but LMP is not detected using our AO relocalization assay. In this case, both LT^S^ and LT^R^ cells would initiate a cell death pathway that involves LMP, but that only cells containing an LT^S^ Nlrp1b amplify and propagate the signal. This would coincide with the observation that cathepsin inhibitors prevent detectable LMP. Therefore, a feedback loop induced first by minor leakage of cathepsins into the cytosol could be amplified by increased inflammasome/caspase-1 activation, Bid cleavage, MOMP and/or activation of unknown signaling molecules.

Our finding that LMP is involved in LT-mediated cell death may explain the activity of various inhibitors reported to protect cells from LT. For example, calpain inhibitors and secretory phospholipase A2 (sPLA2) inhibitors, both of which protect against LMP [80]–[82], also protect cells from LT-induced pyroptosis [49], [83]. In addition, antioxidants such as N-acetyl-cysteine are potent inhibitors of LMP and prevent LT-induced release of IL-1β, a downstream product of inflammasome activation [17], [84], [85]. Finally, heat shock provides strong protection against LT-mediated pyroptosis through an unknown mechanism [54], and Hsp70 protects from both LMP [86]–[88] and MOMP [88]–[93] and is capable of reducing cellular damage associated with these death pathways. Heat shock leads to increased levels of Hsp70 and therefore, Hsp70 may play a substantial role in mediating heat shock-induced resistance to LT. Of note, all proteomic studies report a change in Hsp70, with one group reporting a decrease in Hsp70 [94], while other groups report an increase in Hsp70 following LT treatment [95]–[97]. Whether Hsp70 overexpression is sufficient to protect against LT-induced LMP and pyroptosis is currently being explored. We also report multiple stress response proteins, specifically those involved in heat shock response, are altered during LT-induced pyroptosis. A heat shock-type response may be a reaction to LT- or pyroptosis-associated cell damage, though this response fails to protect cells from major LMP and cytolysis induced by the toxin dose used in *in vitro* studies.

Inflammasome activation and pyroptosis has broad biological significance. While caspase-1 activation protects the host from various pathogens during infection, excessive caspase-1 activation contributes to various inflammatory disorders and septic shock [3], [7], [8]. The control of caspase-1 activation and pyroptosis is an attractive target for protecting cells from both microbial and autoinflammatory attack. Our data support a role for lysosomal cathepsins in NLR-mediated pyroptosis. CtsB is recently implicated in activation of NLRP3-inflammasomes by crystalline structures [77], [78] and in caspase-1 cleavage [76]. Here we show that lysosomal damage and release of cathepsins are central to pyroptosis initiated by LT.

## Materials and Methods

### Cell Culture and Reagents

RAW 264.7 cells and J774A.1 were cultured in DMEM (Cellgro, Mediatech, Inc cat#10-017-CV) and IC-21 cells were cultured in RPMI. Cell lines were obtained from ATCC. Femur exudates from C57Bl/6 or C57BL/6*^Nlrp1b(129S1)^* transgenic animals were cultured for 7 days in DMEM both supplemented with 10% fetal bovine serum (Atlanta, cat#S11550), 1% penicillin/streptomycin/glutamine (Gibco), 2% 14–22 conditioned media and incubated in a 5% CO_2_ humidified incubator at 37°C. BMDMs from three C57BL/6*^Nlrp1b(129S1)^* or C57BL/6 littermate controls were used as replicates for each experiment. Intoxication medium consisted of DMEM containing 25 mM Hepes (Cellgro, Mediatech, Inc. cat#15-018-CV), supplemented with 10% FBS and 1% PSG. PA, LF and LF-H719C were produced and purified as previously described [98]. CA074 (N-1475) and CA074Me (N-1660) were purchased from Bachem (Torrance, CA). LPS was from *Escherichia coli*. Z-FA-FMK (cat# 342000) was purchased from Calbiochem.

### Acridine orange relocation assay

Cultured cells (5×10^5^ per well) were seeded in 6-well plates in DMEM the night before intoxication. The next morning, media was replaced with intoxication media containing 5 µg/ml acridine orange (Calbiochem, cat# 113000) for 15 min under otherwise standard culture conditions. Wells were then rinsed twice with intoxication media and 1 µg/mL PA and 1 µg/mL LF (unless otherwise stated) was added to LT-treated cells. Cells were detached by scraping with a rubber policeman, collected by pelleting at 5,000× g and washed three times with PBS (1 mL). PBS with 1% formaldehyde (300 µL) was used to fix cells and samples were subjected to flow cytometric assessment of red (FL3 channel) and green (FL1 channel) AO fluorescence using a Becton Dickinson FACSCalibur Analytic Flow cytometer. Analysis was performed using FLOWJO flow cytometry analysis software (Tree Star, Inc., Ashland, Oregon).

### Lysotracker staining and imaging

RAW 264.7 cells (1×10^5^ per well) were seeded on 12 mm poly-D-lysine coverslips (BD Biosciences cat# 354086) within 12-well plates the day before experiment. DMEM containing 50 nM Lysotracker Red DND-99 (Invitrogen-Molecular Probes) was added to cells for 90 min under normal growth conditions. Cells were then washed twice with PBS, followed by addition of intoxication media alone or containing 1 µg/mL of LF and 1 µg/mL of PA. Coverslips were removed from dish and live cells were imaged in PBS containing 2 mM MgCl_2_ using an inverted Nikon Eclipse TE300 fluorescence microscope.

### Cytotoxicity assay

For 384-well plate format, cells were seeded at 2×10^3^ cells per well in white-bottom plates. Toxin and/or inhibitors were added to a total of 60 µL of total media and incubated for the time indicated. Experiments were halted with addition of 20 µL of ATP-lite (PerkinElmer, Waltham, Massachusetts) and luminescence was measured using Victor 3V (PerkinElmer) plate reader. Luminescence data is obtained in relative light units (RLU). Background luminescence was subtracted from all samples.

### Cytosolic ctsB activity assay

Cultured cells (6×10^6^) were seeded in 10 cm dishes and treated with 400 ng/mL PA and 300 ng/mL of LF. After 90 minutes, Ak-EVD-AMK (625 nM for cytosolic probing; 5 or 10 µM for total cellular ctsB probing) or DMSO was added to intoxication media for an additional 30 min. The cells were then collected by gentle scraping, pelleted by centrifugation at 2,000× g, and washed twice with PBS (1 mL). Cell pellets were resuspended in 100 µL of ice-cold NP-40/TEA lysis buffer (1% NP-40, 50 mM triethanolamine (TEA), 150 mM NaCl, pH 7.4, with Complete Mini protease inhibitor cocktail (Roche Biosciences, Indianapolis, IN)) for 30 min and then centrifuged at 4°C for 10 min at 13,200× g. Post-nuclear supernatants were collected and protein concentration was determined using Bio-Rad Protein Assay (cat# 500-0001). NP-40/TEA lysis buffer was added to lysates to equate experimental sample volumes and cycloaddition reactions were performed as follows: Azido-rhodamine tag (100 µM, 5 mM stock in DMSO) was added, followed by 1 mM TCEP (50 mM stock in H_2_O) and 100 µM triazole ligand (1.7 mM stock in DMSO:t-butanol 1∶4). The samples were gently vortexed and 1 mM CuSO4 (50 mM stock in H2O) was added. Samples were vortexed again and allowed to react at RT for 1 h. Reactions were terminated by addition of ice-cold acetone (1 mL), incubated at −20°C for 20 min and centrifuged at 13,200× g 4°C for 30 min to precipitate proteins. The supernatants were carefully decanted and the resulting pellets dried at RT for 5 min to remove excess acetone. The protein pellet was subsequently resuspended in 2X SDS-protein reducing buffer and boiled for 10 min. Proteins were then separated by SDS-PAGE and analyzed by in-gel fluorescent scanning using a Typhoon scanner (GE Healthcare; excitation at 532 nM, emission at 580 nM).

### Immunoblotting

Cells were lysed in 1% Triton X-100 buffer (150 mM NaCl; 50 mM Tris-HCl, pH 8; 0.1% SDS; 1% Triton X-100; 5 mM MgCl_2_) with Complete Mini protease inhibitor cocktail (Roche Biosciences), incubated on ice for 30 min, spun down at 13,200× g for 10 min unless otherwise noted. Post-nuclear supernatant protein concentration was determined using Bio-Rad Protein Assay. Proteins were denatured by addition of 6X reducing SDS-protein loading buffer and boiled for 10 min. Samples were vortexed, spun down at 13,200× g for 10 minutes and separated by SDS-PAGE (10% gel for MEK2 and ctsB; 15% gel for Bid/tBid) followed by transfer to PVDF. Membranes were blocked with 5% nonfat dried milk in TBST (50 mM Tris, 150 mM NaCl, 0.5% Tween 20, pH 7.6) for 1 hr at RT, then incubated with primary antibody in 5% nonfat dried milk in TBST for 1 h at RT or overnight at 4°C. Membranes were washed 3x with TBST for 10 min and either developed or probed with secondary antibody (goat anti-rabbit 1∶10,000, Sigma Aldrich) followed by washing 3x with TBST for 20 min. Membranes were developed using ECL reagents (ImmunoStar, BioRad) and autoradiography film (HyBlot, Danville Scientific). To ascertain levels of endogenous proteins, SDS-PAGE gels were stained with coomasie and photographed or membranes were probed with anti-tubulin antibody (T5168 from Sigma Aldrich) followed by goat anti-mouse IgG (cat# 28173 from AnaSpec, Inc. San Jose, CA). Anti-Bid antibody (# 2003), anti-Hsp70 antibody (#4872), anti-eEF2 (#2332) were purchased from Cell Signaling Technology; anti-Bag-1 (C16) antibody (DB004) from Delta Biolabs, Gilroy, CA, anti-MAPRE1 (EB1, H-70: sc-15347) antibody and MEK-2 (N-20): sc-524 from Santa Cruz Biotechnology.

### 2D-DIGE

Two dimensional difference gel electrophoresis (2D-DIGE) was performed at Applied Biomics, Hayward, CA. Cells were seeded the previous day at 10^7^ cells per 10 cm plate. Cells were treated with either PA (1 µg/mL) or PA and LF (1 µg/mL each) for 30 or 40 min. Timing of sample collection was carefully calibrated by western blot analysis of MEK1 and MEK2 cleavage, confirming LF internalization and activity by 20 min. Following LT-challenge, cells were collected by gentle scraping, washed 3x with PBS, pelleted at 2,000× g for 5 min and pellets were flash-frozen in ethanol-dry ice bath and shipped to shipped to Applied Biomics. Proteins were extracted and labeled with either Cy3 or Cy5. Isoelectric focusing in the first dimension was carried out at pH 3–10, and size-based separation in the second dimension was performed using a 9–12% linear gradient SDS-PAGE. Proteomic changes were detected using DeCyder software and spots were cut out at Applied Biomics and mailed to UCLA. Spots were digested with trypsin and subjected to LC-MS/MS (UCLA) using either a QqTOF instrument or nanospray. Peptide sequencing was accomplished with nanoflow high performance liquid chromatography system (LC Packings, Sunnyvale, CA, USA) with a nanoelectrospray (nano-ESI) interface (Protana, Odense, Denmark) and an Applied Biosystems/Sciex QSTAR XL (QqTOF) mass spectrometer (Foster City, CA). The samples were first loaded onto a LC Packings PepMap C18 precolumn (150 µm×3 mm; particle size 5 µm) and washed for two minutes with the loading solvent, 0.1% formic acid. The samples were then injected into a LC Packings PepMap C18 column (75 µm×150 mm; particle size 5 µm) for nano-LC separation at a flow rate of 220 nL/min. For each LC-MS/MS run, typically 6 µL sample solution was loaded to the precolumn first and washed with the loading solvent of 0.1% FA. The eluents used for the LC were (A) 0.1% formic acid and (B) 95% ACN/5% H_2_O/0.1% FA. The following gradient was used: 6% B to 24% B in 18 min, 24% B to 36% B in 6 min, 36% B to 80% B in 2 min and stayed at 80% B for 8 min. The column was finally re-equilibrated with 6% B for 16 min before the next run.

A New Objective (Woburn, MA) PicoTip tip (i.d. 8 µm) was used for spraying with the voltage set at 1750 V. Peptide product ion spectra were automatically recorded during the LC-MS runs by the information-dependent analysis (IDA) on the mass spectrometer. Argon was employed as the collision gas. Collision energies for maximum fragmentation were automatically calculated using empirical parameters based on the charge and mass-to-charge ratio of the peptide. Protein identifications were determined using Mascot database search algorithm (Matrix Science).

## Supporting Information

Figure S1 AO relocalization in ungated cells contain a low red, low green subpopulation. C57BL/6*^Nlrp1b(129S1)^* BMDMs (B6 Tg^+^) or littermate controls (B6) were analyzed for changes in AO fluorescence as in Figure 1B, except cells were not gated via forward and side scatter for normal cell morphology. Density plot represents BMDMs from one of three C57BL/6*^Nlrp1b(129S1)^* or C57BL/6 littermate controls that were tested with similar results for each.
(1.19 MB TIF)

Figure S2 RAW 264.7 and IC-21 cells display a similar phenotype as BMDMs in AO relocalization. (A) RAW 264.7 cells pretreated with AO for 20 minutes were treated with LT (500 ng/mL LF and 1 µg/mL PA) or left untreated (NT) for 3 to 4 hours. Cells were collected for flow cytometry and analyzed as in Figure 1A. Results represent duplicate samples from two independent experiments. Error bars represent standard error. (B) RAW 264.7 were heat shocked at 42°C (RAW + HS) or left untreated (RAW) for 15 min prior to addition of AO, followed by LT (3 µg/mL LF and 1 µg/mL of PA) for 75 and 90 min. Cells were analyzed for changes in AO fluorescence as in Figure 1A. Density plot represents one of three independent experiments with similar results.
(1.32 MB TIF)
