# Supplementary figures and images for: Anthrax Lethal Toxin Induced Lysosomal Membrane Permeabilization and Cytosolic Cathepsin Release Is Nlrp1b/Nalp1b-Dependent
Source: PLoS One. 2009 Nov 18;4(11):e7913. doi: 10.1371/journal.pone.0007913 (PMC2775945; doi:10.1371/journal.pone.0007913)

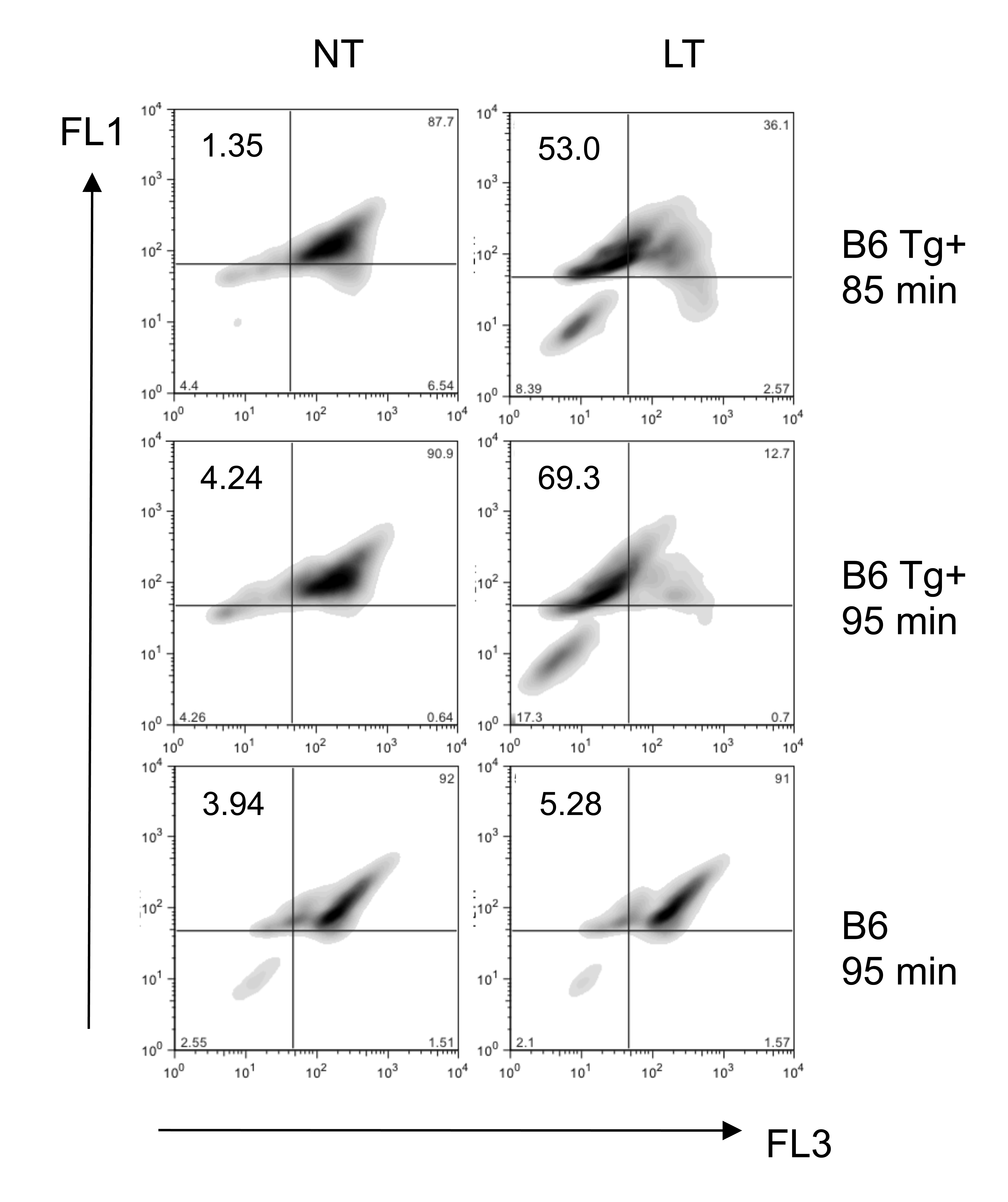

Supplement: Figure S1 — AO relocalization in ungated cells contain a low red, low green subpopulation. C57BL/6Nlrp1b(129S1) BMDMs (B6 Tg+) or littermate controls (B6) were analyzed for changes in AO fluorescence as in Figure 1B, except cells were not gated via forward and side scatter for normal cell morphology. Density plot represents BMDMs from one of three C57BL/6Nlrp1b(129S1) or C57BL/6 littermate controls that were tested with similar results for each. (1.19 MB TIF) [file pone.0007913.s001.tif]

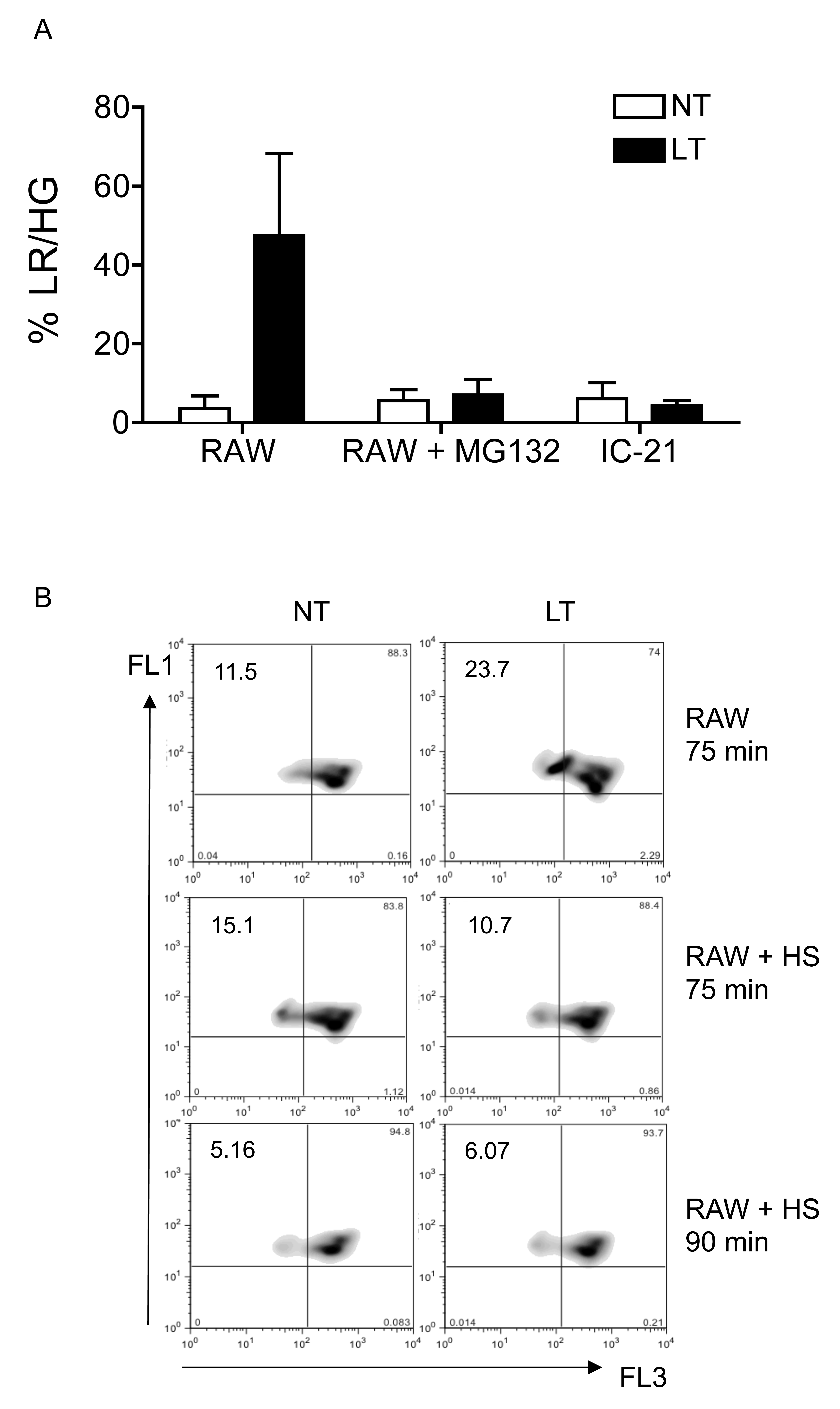

Supplement: Figure S2 — RAW 264.7 and IC-21 cells display a similar phenotype as BMDMs in AO relocalization. (A) RAW 264.7 cells pretreated with AO for 20 minutes were treated with LT (500 ng/mL LF and 1 µg/mL PA) or left untreated (NT) for 3 to 4 hours. Cells were collected for flow cytometry and analyzed as in Figure 1A. Results represent duplicate samples from two independent experiments. Error bars represent standard error. (B) RAW 264.7 were heat shocked at 42°C (RAW + HS) or left untreated (RAW) for 15 min prior to addition of AO, followed by LT (3 µg/mL LF and 1 µg/mL of PA) for 75 and 90 min. Cells were analyzed for changes in AO fluorescence as in Figure 1A. Density plot represents one of three independent experiments with similar results. (1.32 MB TIF) [file pone.0007913.s002.tif]
